# The association between thyroid-stimulating hormone and thyroid nodules, goiter and thyroid antibody positivity

**DOI:** 10.3389/fendo.2023.1204552

**Published:** 2023-10-02

**Authors:** Shaohan Li, Wenxing Guo, Qi Meng, Mei Zhu, Hongyan Wei, Fengying Ji, Long Tan, Wanqi Zhang

**Affiliations:** ^1^ Department of Nutrition and Food Hygiene, School of Public Health, Tianjin Medical University, Tianjin, China; ^2^ Department of Endocrinology and Metabolism, Tianjin Medical University General Hospital, Tianjin, China; ^3^ Qingdao Municipal Center for Disease Control and Prevention, Qingdao, China; ^4^ Qingdao Institute of Preventive Medicine, Qingdao, China; ^5^ Tianjin Key Laboratory of Environment, Nutrition and Public Health, Tianjin, China; ^6^ Tianjin Key Laboratory of Hormones and Development (Ministry of Health), Tianjin, China

**Keywords:** thyroid-stimulating hormone, thyroid dysfunction, thyroid nodules, goiter, thyroid volume, thyroid antibody positivity

## Abstract

**Background:**

The relationship between normal thyroid-stimulating hormone (TSH) levels and thyroid disease in adults remains controversial. This study aimed to investigate the correlation between serum TSH levels, particularly those falling within the normal range, and thyroid diseases in Chinese adults, including thyroid nodules (TN), goiter (GR), and thyroid antibody positivity.

**Materials and methods:**

This research was a cross-sectional study conducted in an adult population in Tianjin, China. Thyroid volume (Tvol) and TN were assessed using thyroid ultrasonography. Fasting venous blood and spot urine samples were collected to evaluate thyroid function and iodine status.

**Results:**

A total of 2460 subjects participated in the survey. The prevalence of thyroid dysfunction was 9.76%, and abnormal TSH levels were found to potentially increase the risk of GR and thyroid antibody positivity in adults. A total of 2220 subjects with TSH within the normal reference range were included in the further study. In these patients, Tvol decreased as TSH levels increased, in both men and women (*P* < 0.0001). Low TSH levels (0.27–1.41 IU/mL) were identified as a risk factor for TN (odds ratio [OR], 1.46; 95% CI: 1.14-1.87) and GR (OR 5.90, 95% CI 2.27-15.3). Upon stratification by sex and age, the risk of TN was found to be higher in women and elderly individuals (≥60 years old), while the risk of GR was found to be higher in men and younger individuals (<60 years old). High TSH levels (2.55–4.2 IU/mL) were identified as a risk factor for thyroid antibody positivity (OR, 1.53; 95% CI: 1.11-2.10). Men and younger individuals with high TSH levels exhibited a higher risk of thyroid antibody positivity.

**Conclusion:**

In adults with normal TSH levels, low TSH levels were associated with an increased risk of TN and GR, whereas high TSH levels were associated with thyroid antibody positivity. The research also suggests that adults whose TSH levels at upper or lower limits of the normal range should be reviewed regularly.

## 1 Introduction

The incidence of thyroid diseases has been increasing in recent years. Thyroid-stimulating hormone (TSH), the most sensitive indicator reflecting thyroid function, is produced by the adenohypophysis and regulated by the hypothalamic-adenohypophysis-thyroid axis. As a stimulator of thyroid growth, TSH is closely associated with the occurrence of various thyroid disorders.

Thyroid nodules (TN) are common thyroid conditions, with a reported prevalence of 4%–7% through palpation, and a prevalence of 13%–67% by high-resolution ultrasonography (1). The American Association of Clinical Endocrinologists recommends that TSH should be reevaluated within 12–24 months based on clinical characteristics and changes in nodules following the initial TSH assessment (2). A study involving 1990 adults in Korea found that TSH level was negatively associated with TN, with patients with TN having significantly higher TSH levels than those without TN (3). In addition, high TSH levels within the normal range may be an early indicator of hypothyroidism and are linked to an increased risk of autoimmune thyroid disease (4). Previous studies have found that thyroid peroxidase antibody (TPOAb) and/or thyroglobulin autoantibody (TgAb) levels significantly increase with increasing TSH levels (5, 6). In China, a study aimed at establishing new reference values for thyroid volume (Tvol) showed that Tvol was negatively correlated with TSH levels. Some studies have also reported that serum TSH levels in adults with goiter (GR) were significantly lower than those in individuals without GR (7). These findings highlight the impact of TSH level fluctuations on the occurrence of thyroid diseases.

TSH levels play a significant role in the development of thyroid diseases through various mechanisms. Clarifying the relationship between TSH levels and thyroid diseases is helpful to the diagnosis of thyroid diseases in clinic. In addition, whether the TSH levels in the normal range can predict the future risk of thyroid diseases is also a topic worthy of attention. This study aims to investigate whether serum TSH levels, especially within the normal range, are associated with common thyroid disorders, including TN, GR, and thyroid antibody positivity.

## 2 Materials and methods

### 2.1 Subjects

A cross-sectional study was conducted from March to October 2015 in Tianjin, China. Participants were chosen through random cluster sampling. To fulfill the research requirements, the subject selection criteria for this study were as follows (1): age > 18 years (2), Han nationality, and (3) residency in Tianjin for >5 years. Subjects who received iodine-containing contrast media or iodine-containing medications within the previous 3 months, or subjects who were undergoing treatment with thyroid hormone or anti-thyroidal drugs for thyroid-related issues were excluded. Pregnant and lactating women were also excluded. The purpose and content of the study were explained in detail to the participating adults. All subjects signed an informed consent. This study was approved by the Ethics Committee of the China Medical University (serial number: IRB [2013]115).

### 2.2 Anthropometric measurements

Each subject was instructed to accurately measure their height and weight. The researchers calculated the body mass index (BMI) for each participant by squaring their weight and dividing it by their height (kg/m2). Furthermore, each subject was required to provide a 5-mL fasting venous blood sample to assess thyroid function, including TSH, free thyroxine (FT4), and serum free triiodothyronine (FT3), as well as TgAb and TPOAb. TSH, FT4, and FT3 levels were determined using the ADVIA Centaur automatic chemiluminescence immunoassay (Siemens Healthcare Diagnostics, Gwynedd, United Kingdom). In cases where TSH levels fell within the normal range, FT3 and FT4 were not measured additionally, whereas if TSH levels were abnormal, FT3 and FT4 were measured. TgAb and TPOAb levels were determined through a chemiluminescence reaction using the IMMULITE 2000 system (Siemens Healthcare Diagnostics Inc, Gwynedd, United Kingdom).

### 2.3 Urinary iodine

Subjects were instructed to collect 5 mL of mid-course spot urine using a plastic centrifuge tube. Urinary iodine was quantified as the urinary iodine concentration (UIC), which was determined through catalytic spectrophotometry with arsenic and cerium. Four levels of freeze-dried human urine reference material (GBW09108l, GBW09110n, GBW09111a, and GBW09112a; National Reference Laboratory for Iodine Deficiency Disorders, Beijing) were run together with each batch of samples, and the average standard iodine concentrations were 68 (range: 59–77) μg/L, 195 (range: 185–205) μg/L, and 558 (range: 541–575) μg/L. The coefficient of variation for UIC ranged from 0.1% to 4.7%.

### 2.4 Thyroid ultrasonography

Tvol was measured by professionally trained operators using a 7.5 MHz transducer (Madison Portable B-SA-600). During the procedure, subjects remained seated with their upper bodies straight and fully exposing their necks. The operator systematically scanned the thyroid gland in an up-and-down, left-to-right fashion, recording the maximum width of the thyroid gland. Subsequently, longitudinal scanning was conducted first on the left side and then on the right side, recording both the maximum depth and maximum length of the thyroid lateral lobe. The unilateral volume was calculated using the formula: Tvol (mL) = 0.479 × width × depth × length (mm)/1000, and the total Tvol was determined as the sum of the left and right thyroid volumes.

### 2.5 Definition and diagnostic

According to the Diagnostic Criteria for Endemic Goiter (WS-2007), Tvol exceeding 25.0 mL for men and 18.0 mL for women was considered indicative of GR. TN have been described as additional abnormalities in ultrasonic structures. The reference range for TSH provided by the Clinical Laboratory of Tianjin Medical University General Hospital was 0.27–4.2 IU/mL. In this study, when serum TSH levels fell within this normal range, the thyroid function of the subjects was deemed normal. In addition, thyroid antibody positivity was defined as having TPOAb > 34 IU/mL and/or TgAb > 115 IU/mL.

### 2.6 Statistical analysis

All statistical analyses were conducted using IBM SPSS Statistics (version 26). The Kolmogorov-Smirnov test was used to test the normality of continuous variables. Non-normally distributed data were presented as median (interquartile range, IQR). Categorical variables were described as ratios and percentages. The Mann–Whitney U test and the Kruskal–Wallis test were used to compare non-normally distributed data. The chi-square test was used to compare the prevalence of TN, GR, and thyroid antibody positivity. Trend tests were performed to evaluate changes in UIC, Tvol, TN, and GR and the antibody positivity rate across the TSH quartiles. Univariate logistic analysis was used to assess whether TSH levels falling below the 25th percentile or above the 75th percentile were associated with thyroid diseases. An interaction test was employed to analyze the interaction between sex, age, and TSH levels on the prevalence of thyroid diseases. *P* < 0.05 was considered to be statistically significant.

## 3 Results

### 3.1 General population characteristics

A total of 2460 subjects were enrolled. The prevalence of thyroid dysfunction was 9.76%, including 16 patients with hyperthyroidism and subclinical hyperthyroidism and 224 patients with hypothyroidism and subclinical hypothyroidism. After excluding 240 subjects with abnormal TSH levels, a total of 2220 adults with normal thyroid function were included for further analysis. The baseline data for the subjects are presented in **Table 1**. There were no significant differences in age distribution between men and women (*P* = 0.48). Men had a higher BMI than women (*P* < 0.0001). The median (IQR) UIC was 135 (81.8-208) μg/L, which indicated adequate iodine nutrition in the population. The median (IQR) Tvol was 9.07 (7.17-11.6) mL. The medians (IQR) of TSH in men and women were 1.81 (1.32–2.39) IU/mL and 2.05 (1.53–2.75) IU/mL, respectively. Women exhibited a higher prevalence of TN and thyroid antibody positivity than men.

**Table 1:** Baseline characteristics of participants with normal TSH level.

Variables	Total (n = 2220)	Men (n = 1221)	Women (n = 999)	*P*
Age, y	41 (29–53)	42(29-53)	40(28-53)	0.48
Height, cm	167(161-174)	173(169-177)	160(157-164)	<0.0001
Weight, kg	68.0(59.0-79.0)	75.0(68.0-84.0)	61.0(55.0-67.0)	<0.0001
BMI, kg/m^2^	24.6(22.0-27.1)	25.4(23.0-27.8)	23.6(21.2-26.2)	<0.0001
UIC, μg/L	135(81.8-208)	139(86.7-209)	126(76.2-206)	0.03
Tvol, mL	9.07(7.17-11.6)	10.2(8.33-12.7)	7.73(6.13-9.95)	<0.0001
TSH, IU/mL	1.90(1.41-2.55)	1.81(1.32-2.39)	2.05(1.53-2.75)	<0.0001
Thyroid nodules, n %	535(24.1)	274(22.4)	261(26.1)	0.04
Goiter, n %	22(1.0)	10(0.8)	12(1.2)	0.36
Thyroid Antibody positivity, n%	230(10.4)	65(5.3)	165(16.5)	<0.0001

BMI, body mass index; UIC, urinary iodine concentration; Tvol, thyroid volume; TSH, thyroid-stimulating hormone.

### 3.2 Prevalence rate of thyroid diseases in quartiles of different TSH

**Figure 1:**
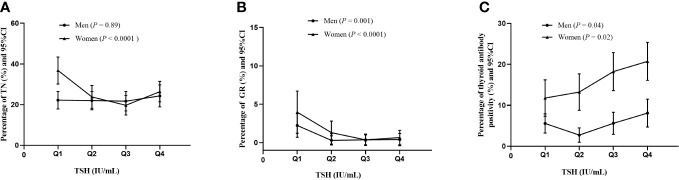
Percentage of thyroid diseases in men and women according to TSH quartiles. TN **(A)**, GR **(B)**, thyroid antibody positivity **(C)**.

The percentages of thyroid diseases across different TSH quartiles are depicted in **Figure 1**. Tvol in men from TSH-Q1 to TSH-Q4 was 11.2 mL, 10.4 mL,9.86 mL, and 8.99 mL (P < 0.0001). Similarly, in women, Tvol was 8.26 mL, 7.91 mL, 7.58 mL, and 7.45 mL (P < 0.0001; **Table 2**). With the increase of TSH levels in men and women, the prevalence of GR had a downward trend. In women, the proportion of TN in the TSH-Q1 group was higher than that in the TSH-Q2 group and TSH-Q3 group. In addition, the prevalence rate of thyroid antibody positivity in TSH-Q4 group was significantly higher than that in TSH-Q2 group in men. With the increase of TSH levels in women, the prevalence of thyroid antibody positivity had an increasing trend. We analyzed the continuous characteristics of the different TSH level groups and presented the corresponding *P* values for trends in **Table 2**.

**Table 2:** Characteristics of men and women subjects between TSH level which were divided into four groups.

Variables	TSH (IU/mL)				P	P for trend
	Q1 0.27-1.41	Q2 1.42-1.90	Q3 1.91-2.55	Q4 2.56-4.20		
Men						
Tvol (ml)	11.2(9.12-13.5)	10.4(8.41-12.8)	9.86(8.30-12.4)	8.99(7.60-11.1)	<0.0001	<0.0001
TN (n, %)	80(22.2)	72(22.0)	62(21.7)	60(24.3)	0.89	0.63
GR (n, %)	8(2.2)	1(0.3)	1(0.4)	1(0.4)	0.001	0.003
Antibody positivity (n, %)	20(5.6)	9(2.7)	16(5.6)	20(8.1)	0.04	0.12
Women						
Tvol (ml)	8.26(6.72-10.8)	7.91(6.19-9.79)	7.58(5.86-9.95)	7.45(5.71-9.43)	<0.0001	<0.0001
TN (n, %)	75(36.8)	54(23.8)	53(19.7)	79(26.4)	<0.0001	0.02
GR (n, %)	8(4.0)	3(1.3)	1(0.4)	2(0.7)	<0.0001	0.001
Antibody positivity (n, %)	24(11.8)	30(13.2)	49(18.2)	62(20.7)	0.02	0.002

Tvol, thyroid volume; TN, thyroid nodules; GR, goiter.

### 3.3 Relationship between abnormal TSH and thyroid diseases

The relationship between abnormal TSH levels and TN, GR, and thyroid antibody positivity was investigated (**Table 3**). We defined the normal TSH range (0.27–4.2 IU/mL) as the reference point. Logistic regression analysis revealed that after adjusting for sex, age, height and weight, TSH < 0.27 IU/mL or TSH > 4.2 IU/mL may elevate the risk of GR and thyroid antibody positivity. Specifically, the risk of GR in adults with TSH < 0.27 IU/mL and TSH > 4.2 IU/mL was 20.2 and 4.36 times higher, respectively, than in individuals with normal TSH levels. Similarly, the risk of thyroid antibody positivity in adults with TSH < 0.27 IU/mL and TSH > 4.2 IU/mL was 7.88 and 6.02 times higher, respectively, than in those with normal TSH levels.

**Table 3:** Logistic analysis of abnormal TSH levels as risk factors for thyroid disease.

Variables	Unadjusted			Adjusted^*^		
	OR	95% CI	*P*	OR	95%CI	*P*
When TSH is < 0.27 IU/mL						
TN	1.43	(0.49-4.14)	0.51	1.86	(0.60-5.75)	0.28
GR	14.2	(3.04-66.1)	0.001	20.2	(4.04-101)	<0.0001
Thyroid antibody positivity	6.73	(2.48-18.2)	<0.0001	7.88	(2.77-22.4)	<0.0001
When TSH is > 4.2 IU/mL						
TN	1.15	(0.84-1.57)	0.37	0.97	(0.70-1.34)	0.85
GR	5.62	(2.74-11.5)	<0.0001	4.36	(2.08-9.12)	<0.0001
Thyroid antibody positivity	7.10	(5.28-9.56)	<0.0001	6.02	(4.43-8.19)	<0.0001

^*^Adjusted for sex, age, height, weight.

TSH, thyroid-stimulating hormone; TN, thyroid nodules; GR, goiter.

### 3.4 Relationship between normal TSH and thyroid diseases

The middle TSH group (with TSH levels ranging from 1.41 to 2.55 IU/mL) was regarded as the reference group. TSH levels below the 25th percentile were categorized as low TSH (TSH < 1.41 IU/mL), whereas levels above the 75th percentile were categorized as high TSH (TSH > 2.55 IU/mL). Regression analysis revealed that both low and high TSH levels can be risk factors for thyroid diseases (**Table 4**). The odds ratio (OR) for TN in adults with lower TSH levels was 1.46 compared to those with higher TSH level (1.41–2.55 IU/mL). Adults with low TSH levels were associated with an increased risk of GR (OR, 5.90; 95% CI, 2.27-15.3). Similarly, the risk of thyroid disease was significantly elevated in individuals with TSH > 2.55 IU/mL, with a significantly increased risk of antibody positivity (OR, 1.53; 95% CI, 1.11-2.10).

**Table 4:** Logistic analysis of TSH < 25 percentile or TSH > 75 percentile as a risk factor for thyroid disease.

Variables	Unadjusted			Adjusted^*^		
	OR	95% CI	*P*	OR	95%CI	*P*
When TSH is < 1.41 IU/mL						
TN	1.36	(1.08,1.72)	0.01	1.46	(1.14-1.87)	0.003
GR	5.36	(2.08-13.8)	<0.001	5.90	(2.27-15.3)	<0.001
Thyroid antibody positivity	0.82	(0.57-1.18)	0.29	0.90	(0.62-1.31)	0.58
When TSH is > 2.55 IU/mL						
TN	1.23	0.97-1.56	0.09	1.16	(0.91-1.49)	0.23
GR	0.00	(0.00-)	0.99	0.00	(0.00-)	0.97
Thyroid antibody positivity	1.71	1.25-2.33	0.00	1.53	(1.11-2.10)	0.01

^*^Adjusted for sex, age, height, weight.

TSH, thyroid-stimulating hormone; TN, thyroid nodules; GR, goiter.

**Figure 2:**
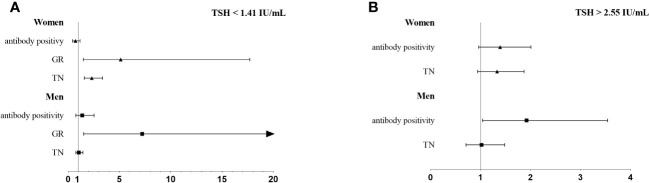
The association of thyroid diseases with TSH < 25 percentile **(A)** or TSH > 75 percentile **(B)** stratified by sex.

**Figure 2** and **Table 5** displays the risk of thyroid diseases with low and high TSH levels stratified by sex and age (<60 years and ≥60 years). On the one hand, TN was significantly associated with low TSH levels in women (OR: 2.30, 95% CI, 1.57-3.36) but not in men. Furthermore, the risk was significantly higher in the ≥60-year-old group than in the <60-year-old group (OR: 1.75, 95% CI: 1.03-2.96 vs. OR: 1.34, 95% CI: 1.02-1.76). However, the interaction between age and low TSH levels was not statistically significant for the risk of TN (*P* for interaction = 0.98). Men or young people (<60 years old) with low TSH levels had a higher risk for GR than women or elderly people (≥60 years old). Considering the interaction of sex or age with low TSH levels, low TSH had no significant effect on GR (*P* for interaction = 0.66, *P* for interaction = 0.57, respectively). On the other hand, in young adults (<60-year-old group), thyroid antibody positivity was significantly correlated with high TSH levels (OR, 1.54; 95% CI, 1.08–2.20), but this association was not observed in older adults (≥60-year-old group). Furthermore, the risk of antibody positivity was notably higher in the men group (OR: 1.92, 95% CI: 1.04–3.54) than in the women group (OR: 1.39, 95% CI: 0.96-2.01), although the interaction between sex and high TSH was not statistically significant (P for interaction = 0.32).

**Table 5:** The association of thyroid diseases with TSH < 25 percentile or TSH > 75 percentile stratified by age.

Variables	Unadjusted			Adjusted		
	OR	95% CI	*P*	OR	95%CI	*P*
When TSH is < 1.41 IU/mL						
< 60						
TN	1.29	0.99-1.69	0.06	1.34	1.02-1.76	0.03
GR	6.66	1.82-24.3	0.004	6.82	1.85-25.2	0.004
Thyroid antibody positivity	0.77	0.50-1.16	0.27	0.88	0.57-1.34	0.54
≥60						
TN	1.78	1.05-3.01	0.03	1.75	1.03-2.96	0.04
GR	4.17	1.02-17.1	0.047	4.24	1.02-17.7	0.047
Thyroid antibody positivity	1.04	0.47-2.27	0.92	1.01	0.45-2.27	0.97
When TSH is > 2.55 IU/mL						
< 60						
TN	1.23	0.93-1.62	0.14	1.19	(0.90-1.58)	0.21
GR	0.00	0.00	0.99	0.00	0.00	0.98
Thyroid antibody positivity	1.74	1.23-2.47	0.002	1.54	1.08-2.20	0.02
≥60						
TN	1.08	0.65-1.78	0.77	1.11	0.67-1.85	0.68
GR	0.00	0.00-	1	0.00	0.00-	1
Thyroid antibody positivity	1.50	0.76-2.98	0.24	1.47	0.73-2.94	0.28

^*^Adjusted for sex, age, height, weight.

TSH, thyroid-stimulating hormone; TN, thyroid nodules; GR, goiter.

## 4 Discussion

Thyroid diseases represent the most prevalent group of disorders within the endocrine system. TSH levels play a significant role in the development of thyroid diseases through various mechanisms. The main cause of GR remains iodine deficiency (8). When dietary iodine intake is insufficient to synthesize thyroid hormones, serum thyroxine reduces, leading to increased TSH release by the pituitary gland. Elevated TSH stimulates the growth and metabolism of thyroid follicular cells, potentially causing GR. In addition, TSH binds to the TSH receptor on thyroid follicular epithelial cells, activates G proteins, and stimulates thyroid hyperplasia through the GS-AC-cAMP signaling system; the development of TN may increase as TSH levels increase. Additional studies have reported higher rates of TPOAb alone, TgAb alone, or at least one antibody positivity in both the low TSH and high TSH values groups than in euthyroid individuals (9). As demonstrated in our study, abnormal TSH levels were associated with an increased risk of GR and antibody positivity in adults. As the primary hormone regulating thyroid function, TSH is the most important indicator for the laboratory diagnosis of thyroid diseases. Therefore, it would also be particularly interesting to explore the future risk of thyroid disease in subjects with currently normal TSH levels. This study focused on the relationship between normal TSH levels and common thyroid diseases.

In this study, the prevalence of TN was 26.1% in women and 22.4% in men, consistent with a report by Chen et al. (10), in which the prevalence rate of TN in Hangzhou in 2013 was 24.1% for men and 34.7% for women (11, 12). In addition, it is well known that women are more likely than men to develop TN, which is consistent with our findings. TSH significantly regulates the growth and differentiation of thyroid cells and may play a direct role in nodule formation (13). Currently, numerous studies have examined the relationship between thyroid function and TN, yet a definitive conclusion remains elusive. For instance, one study on the elderly concluded that TN in this age group had no correlation with laboratory thyroid function indicators (14). Polyps et al. pointed out that within the normal range, an increase in TSH levels is associated with an elevated risk of malignant tumors in TN (15). Wu et al. reported the same conclusion (16). In contrast, some studies have reported that TSH levels were negatively correlated with TN (3, 17). In this study, TSH levels and the prevalence of TN were analyzed in individuals with normal thyroid function, which revealed that TN prevalence was significantly higher in the TSH-Q1 group (0.27–1.41 IU/mL) than in other groups. This observation was consistent with the findings of Wu et al. (18), which found a correlation between the prevalence of TN and declining TSH levels, even after accounting for factors such as age, sex, smoking, alcohol consumption, and medication use. Moreover, we discovered that a TSH level of 0.27–1.41 IU/mL (low TSH level) was a risk factor for TN. A study on TN in Cyprus women showed that the median serum TSH level was lower in women with nodules than in those without nodules (1.4 mIU/L and 1.7 mIU/L, respectively), and the median serum TSH remained within the normal range (17). In addition, our study indicated that the prevalence of TN increased significantly only in the women’s group among those with low TSH levels, which is consistent with the findings of a Beijing adult study, indicating that women are independent risk factors for TN (19). Estrogen may play a role in the formation of TN, as it can influence thyroid tissue and stimulate the production of TSH. Estrogen receptors are expressed in both normal and tumor thyroid tissue, suggesting that estrogen may contribute to thyroid cell growth and nodule formation (19). We made a stratified analysis of men and women, with age of 50 as the cutoff point for stratification. Similar to the above results, the risk of TN only increased in women (**Supplementary Tables 1**, **2**). In addition, we found that in populations with low TSH levels, the risk of TN was notably higher in adults aged >60 years than in those aged <60 years. However, regarding the prevalence of TN, there was no significant interaction between age and low TSH levels. This indicates that TSH is valuable in predicting the nature of TN, but further investigation is needed. Furthermore, we found that Tvol decreased with increasing TSH levels in both men and women, even within the normal TSH range. While some studies have demonstrated a clear correlation between TSH and Tvol (20, 21), others have not observed this correlation (22). In a cross-sectional and longitudinal analysis of obese children, GR was not associated with thyroid function parameters (23). In contrast, our study revealed that in addition to the negative correlation between TSH and Tvol, the risk of GR increased when TSH levels within the normal range were <1.41 IU/mL, with a higher risk in men than in women and in the young than in the elderly. A Lithuanian study reported a negative association between Tvol and TSH, regardless of the presence of TN, with patients with GR having lower TSH levels than those without GR (24). Other studies have also found a negative association between TSH levels and GR, with a reduced risk of GR at higher TSH levels (25, 26). These findings are consistent with the findings of our study. Available data suggest that women are more prone to developing GR than men, especially in iodine-deficient areas (27, 28). This difference may be attributed to the hormonal status of women, wherein estrogen downregulates the sodium/iodine symporter (29). Some authors have reported that sex-related differences become apparent after puberty, which indicated that sex hormones may play a role in Tvol (30). After stratified analysis of men and women with age of 50 as the cutoff point for stratification, it was found that the risk of GR was obviously increased when TSH was at a low level (0.27-1.41 IU/mL) for women over 50 years old (**Supplementary Tables 1**, **2**). Advanced age has been identified as an independent risk factor for GR (31), with one study on factors related to the prevalence of GR in Turkish populations revealing that the prevalence of GR increased with age and reached its peak (38.4%) in those aged >70 years (25). A Danish study showed that the prevalence of GR increased with age but plateaued at 40–45 years old (29).

The presence of thyroid antibodies may serve as an early indicator of thyroid disease, particularly autoimmune thyroid disease (4). TPOAb and/or TgAb have been shown to significantly increase with increasing TSH levels and have been associated with an elevated risk of hypothyroidism (5, 6). Another study found that there was a positive correlation between TPOAb levels and TSH, suggesting that TPOAb and TSH levels may predict hypothyroidism risk, even when TSH levels fall within the normal laboratory reference range (32). In this study, TSH > 2.55 IU/mL within the normal range were associated with an increased risk of antibody positivity. Earlier studies by Jensen et al. (33) and Hollowell et al. (34) linked the presence of TPOAbs not only to a higher frequency of elevated TSH levels outside the normal range but also to a trend toward higher TSH levels within the normal range. This indicates that the presence of TPOAbs necessitate a compensatory increase in TSH levels to maintain normal thyroid function (32). Our study further revealed that while the incidence of thyroid antibody positivity was higher in women than in men, logistic regression analysis showed that men with high TSH levels were at a greater risk of antibody positivity than women. Furthermore, our results demonstrated that high TSH levels in young adults increased the risk of antibody positivity. A Dutch study reported that the risk of positive TPOAb levels decreased with increasing age (35). Several studies have reported that various subsets of T and B lymphocytes decrease with age (36–39), which may lead to a decrease in thyroid lymphocyte infiltration, subsequently lowering the production of TPOAb in older individuals.

We conducted an analysis based on a large-scale epidemiological survey to analyze the association of abnormal and normal TSH levels with common thyroid diseases. This study shows that abnormal TSH levels may increase the risk of GR and thyroid antibody positivity. In addition, our research results show that the normal TSH levels can also predict the future risk of thyroid diseases. This also suggests that adults with TSH at higher or lower levels within the normal range, especially those at upper or lower limits of the normal range, should be reviewed regularly. Nevertheless, several limitations need to be acknowledged. First, not all subjects underwent laboratory testing for FT4 and FT3; only adults with abnormal TSH levels were tested. Second, comorbidities and concomitant medications are common among the elderly. Certain drugs, such as estrogen, aspirin, non-steroidal anti-inflammatory drugs, and glucocorticoids, can impact thyroid hormones. Finally, this study was cross-sectional and did not account for time. Therefore, we cannot infer a causal relationship between TSH and TN, GR, and antibody positivity in the euthyroid population. More longitudinal cohort studies are needed to further investigate these associations.

## 5 Conclusion

In summary, our study suggests that lower TSH levels (0.27-1.41 IU/mL) were associated with an increased risk of TN and GR, while higher TSH levels (2.55-4.20 IU/mL) were associated with thyroid antibody positivity. A larger sample size cohort study is needed to investigate the relationship between normal TSH levels and thyroid diseases. In addition, the research also suggests that adults whose TSH levels at upper or lower limits of the normal range should be reviewed regularly.

## Data availability statement

The raw data supporting the conclusions of this article will be made available by the authors, without undue reservation.

## Ethics statement

The studies involving humans were approved by the Ethics Committee of the China Medical University. The studies were conducted in accordance with the local legislation and institutional requirements. The participants provided their written informed consent to participate in this study.

## Author contributions

FJ, LT and WZ designed the research. SL, WG and QM conducted the research and collected the data. MZ and HW performed the laboratory analysis. SL and WG performed the statistical analysis. SL and WG wrote the paper. All authors have read and approved the final manuscript.
