# Supplementary material for: The association between thyroid-stimulating hormone and thyroid nodules, goiter and thyroid antibody positivity
Source: Front Endocrinol (Lausanne). 2023 Oct 2;14:1204552. doi: 10.3389/fendo.2023.1204552 (PMC10577406; doi:10.3389/fendo.2023.1204552)
Supplement: Supplementary file 1 [file Table_1.docx]

***Supplementary Material***

Supplementary Table 1 The association of thyroid diseases with TSH < 25 percentile or TSH > 75 percentile in men with age of 50 as the cutoff point for stratification

| Variables | Unadjusted | | |  | Adjusted^*^ | | |
| --- | --- | --- | --- | --- | --- | --- | --- |
|  | OR | 95% CI | *P* |  | OR | 95%CI | *P* |
| **When TSH is < 1.41 IU/mL** |  |  |  |  |  |  |  |
| **<50** |  |  |  |  |  |  |  |
| TN | 0.97 | 0.63-1.48 | 0.88 |  | 0.91 | 0.59-1.41 | 0.69 |
| GR | 0.00 | 0.00- | 0.99 |  | 0.00 | 0.00- | 0.99 |
| Thyroid antibody positivity | 1.52 | 0.73-3.17 | 0.26 |  | 1.44 | 0.68-3.02 | 0.34 |
| **≥50** |  |  |  |  |  |  |  |
| TN | 1.10 | 0.69-1.81 | 0.71 |  | 1.23 | 0.73-2.06 | 0.44 |
| GR | 2.59 | 0.43-15.8 | 0.30 |  | 2.34 | 0.37-14.7 | 0.36 |
| Thyroid antibody positivity | 1.14 | 0.39-3.30 | 0.81 |  | 1.27 | 0.42-3.78 | 0.67 |
| **When TSH is > 2.55 IU/mL** |  |  |  |  |  |  |  |
| **<50** |  |  |  |  |  |  |  |
| TN | 1.05 | 0.64-1.71 | 0.85 |  | 0.97 | 0.58-1.62 | 0.92 |
| GR | 0.00 | 0.00- | 0.99 |  | 0.00 | 0.00- | 0.98 |
| Thyroid antibody positivity | 1.42 | 0.60-3.40 | 0.42 |  | 1.36 | 0.56-3.28 | 0.49 |
| **≥50** |  |  |  |  |  |  |  |
| TN | 1.04 | 0.62-1.76 | 0.87 |  | 1.02 | 0.60-1.75 | 0.93 |
| GR | 0.00 | 0.00- | 1.00 |  | 0.00 | 0.00- | 0.98 |
| Thyroid antibody positivity | 2.81 | 1.14-6.95 | 0.02 |  | 2.79 | 1.13-6.91 | 0.03 |

^*^Adjusted for sex, age, height, weight.

TSH, thyroid-stimulating hormone, TN, thyroid nodules, GR, goiter.

Supplementary Table 2 The association of thyroid diseases with TSH < 25 percentile or TSH > 75 percentile in women with age of 50 as the cutoff point for stratification

| Variables | Unadjusted | | |  | Adjusted^*^ | | |
| --- | --- | --- | --- | --- | --- | --- | --- |
|  | OR | 95% CI | *P* |  | OR | 95%CI | *P* |
| **When TSH is < 1.41 IU/mL** |  |  |  |  |  |  |  |
| **<50** |  |  |  |  |  |  |  |
| TN | 2.17 | 1.36-3.47 | 0.001 |  | 2.33 | 1.43-3.80 | 0.001 |
| GR | 0.00 | 0.00- | 0.99 |  | 0.00 | 0.00- | 0.99 |
| Thyroid Antibody positivity | 0.64 | 0.35-1.18 | 0.15 |  | 0.64 | 0.35-1.19 | 0.16 |
| **≥50** |  |  |  |  |  |  |  |
| TN | 2.40 | 1.31-4.39 | 0.01 |  | 2.38 | 1.29-4.39 | 0.01 |
| GR | 4.06 | 1.10-14.9 | 0.03 |  | 3.74 | 1.00-13.9 | 0.05 |
| Thyroid Antibody positivity | 0.84 | 0.37-1.93 | 0.69 |  | 0.82 | 0.36-1.90 | 0.65 |
| **When TSH is > 2.55 IU/mL** |  |  |  |  |  |  |  |
| **<50** |  |  |  |  |  |  |  |
| TN | 1.65 | 1.06-2.55 | 0.02 |  | 1.70 | 1.08-2.66 | 0.02 |
| Thyroid Antibody positivity | 1.44 | 0.93-2.24 | 0.10 |  | 1.44 | 0.92-2.24 | 0.11 |
| **≥50** |  |  |  |  |  |  |  |
| TN | 0.99 | 0.57-1.71 | 0.96 |  | 0.99 | 0.57-1.73 | 0.97 |
| Thyroid Antibody positivity | 1.27 | 0.65-2.49 | 0.48 |  | 1.30 | 0.66-2.56 | 0.45 |

^*^Adjusted for sex, age, height, weight.

TSH, thyroid-stimulating hormone, TN, thyroid nodules, GR, goiter.
